# A triazine-based Ni(II) PNP pincer complex as catalyst for Kumada–Corriu and Negishi cross-coupling reactions

**DOI:** 10.1007/s00706-016-1878-4

**Published:** 2016-12-09

**Authors:** Mathias Mastalir, Karl Kirchner

**Affiliations:** Institute of Applied Synthetic Chemistry, Vienna University of Technology, Getreidemarkt 9, 1060 Vienna, Austria

**Keywords:** Metal complexes, Pincer ligands, Homogeneous catalysis, Cross-coupling, Nickel

## Abstract

**Abstract:**

Kumada–Corriu and Negishi cross-coupling reactions, catalyzed efficiently by a Ni(II) PNP pincer complex containing a triazine backbone, are described. The catalyst is able to react with both activated and inactivated aryl halides including chlorides as well as phenol derivatives such as tosylates and mesylates to give the corresponding cross-coupling products in good to excellent isolated yields. A high diversity of substrates was tested under moderate conditions for both types of reactions.

**Graphical Abstract:**

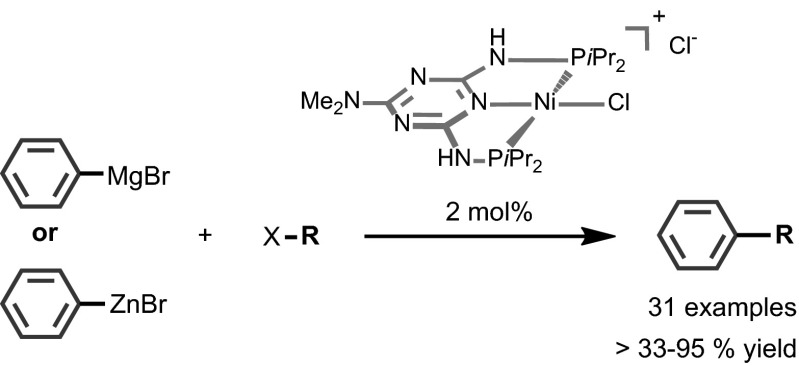

**Electronic supplementary material:**

The online version of this article (doi:10.1007/s00706-016-1878-4) contains supplementary material, which is available to authorized users.

## Introduction

Transition metal catalyzed cross-coupling reactions have been extensively investigated and widely applied in organic synthesis [1]. Among such coupling reactions, Kumada–Corriu coupling reaction, which was reported independently in 1972 by the groups of Makoto Kumada [2] and Robert Corriu [3] as well as the Negishi cross-coupling, reported in 1977 by Ei-ichi Negishi [4], play an important role. These reactions involve the coupling of organomagnesium and organozinc reagents, respectively, with organic halides or pseudohalides and have become one of the most important and prevalent methods for the construction of carbon–carbon bonds as shown in Scheme 1 [5–12]. An advantage over other cross-coupling reactions is the high reactivity of organometallic reagents, their cost efficiency, and their applicability for industrial relevant reactions [13–16]. Although palladium catalysts still dominate the field of cross-coupling reactions, the use of nickel catalysts has become increasingly important [17–19]. Nickel is more abundant and less expensive as compared to palladium and thus preferable in terms of sustainability and economic viability [20]. In particular, Ni pincer complexes have been rarely used as catalyst for cross-couplings [17, 21–23]. Hu and co-workers applied the first anionic Ni NNN pincer system for Kumada–Corriu cross-coupling reactions [24, 25].

**Figure:**
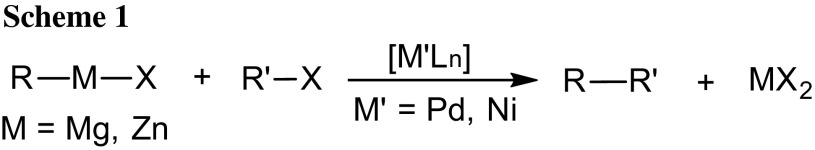

We report here the application of air-stable and well-defined Ni(II) PNP pincer complexes based on the triazine scaffold as catalysts for the Kumada–Corriu and Negishi cross-coupling reactions of several sp^2^ and sp^3^ halides and pseudohalides with different Grignard and organozinc reagents (Scheme 2). Related V, Cr, and Mn PNP pincer complexes reported previously afforded under Kumada–Corriu conditions only homo-coupled products [26]. Triazine-based Ni PNP pincer complexes were recently utilized as catalysts for the Suzuki–Miyaura cross-coupling of aryl and alkyl halides [27], while Fe PNP triazine complexes were used as catalysts for the alkylation of amines by alcohols [28]. Moreover, this type of ligands was also successfully applied by Kempe and co-workers in the cobalt and manganese catalyzed hydrogenation of carbonyl compounds [29, 30] as well as for the alkylation of amines, unactivated amides, and esters with alcohols [31, 32]. These recent examples emphasize the potential of triazine-based PNP pincer complexes for catalytic applications.

**Figure:**
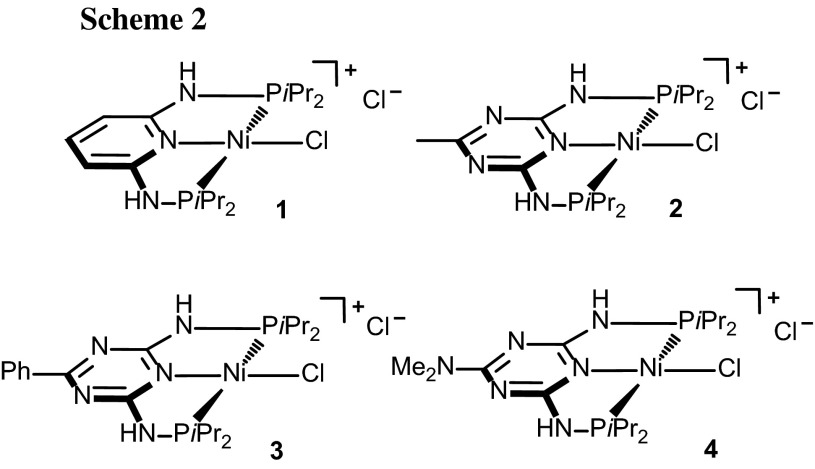

## Results and discussion

The precatalysts **1–4** (Scheme 2) were prepared by treatment of NiCl_2_·6H_2_O with the respective PNP ligands as reported previously [27, 32] and are thermally robust and air-stable compounds. The reaction of 4-bromotoluene with phenyl magnesium bromide and phenyl zinc bromide in THF at room temperature and 60 °C, respectively, for 6 h was investigated with a catalyst loading of 2 mol% to identify the most efficient catalyst for these coupling reactions (Tables 1, 2). In the case of the Negishi coupling, LiCl was added to enhance the reactivity of the phenyl zinc bromide following a procedure developed by Knochel and co-workers [33]. In both type of reactions complex **4** turned out to the best catalyst affording 96 and 94% isolated yields of 4-methylbiphenyl (Tables 1, 2, entry 4), while the pyridine-based complex **1** was the least active one (Tables 1, 2, entry 1). Moreover, based on a negative mercury drop test, we believe that the pincer complexes are the direct catalyst of the reactions and that the reactions proceed in a homogeneous fashion.

**Table 1:** Catalyst screening for the Kumada–Corriu cross coupling

Entry	Catalyst	Base	Yield/%^a^
1	**1**	PhMgBr	47
2	**2**	PhMgBr	91
3	**3**	PhMgBr	86
4	**4**	PhMgBr	96

4-Bromotoluene (1 mmol) and catalyst (2 mol%) in 3 cm^3^ THF, PhMgBr (1.3 mmol) was added and stirred at r.t. for 6 h

^a^Yield of pure isolated product after column chromatography

**Table 2:** Catalyst screening for the Negishi cross coupling

Entry	Catalyst	Base	Yield/%^a^
1	**1**	PhZnBr	52
2	**2**	PhZnBr	92
3	**3**	PhZnBr	90
4	**4**	PhZnBr	94

4-Bromotoluene (1 mmol) and catalyst (2 mol%) in 3 cm^3^ THF, PhZnBr (0.5 M in THF, 1.3 mmol) and LiCl (1.3 mmol) were added at r.t. and the solution was stirred at 60 °C for 6 h

^a^Yield of pure isolated product after column chromatography

Accordingly, the potential of **4** as catalyst was evaluated for the coupling of various aryl and heteroaryl halides (including chlorides) and pseudohalides (triflates, tosylates) with phenyl magnesium bromide and phenyl zinc bromide, respectively. The results of the couplings catalyzed by complex **4** are summarized in Table 3. In general, good to excellent isolated yields were observed for most substrates containing electron donating groups (OMe, Table 3, entries 8–13) or electron withdrawing groups (acyl, Table 3, entry 14). Also heteroaryl halides, based on benzoxazole, thiazole, pyridine, and thiophene, afforded good yields (Table 3, entries 15–24). Moreover, we tested the reactivity of non-activated primary and secondary aliphatic triflates (Table 3, entries 28–30). This reaction proceeds in good to moderate isolated yields in the case of the Negishi coupling (Table 3, entries 28 and 30). With butyl triflate under Kumada–Corriu conditions, a lower yield was observed (Table 3, entry 29). The lower yield may be due to elimination reactions of the alkyl chain under basic conditions. Finally, the vinylation of 4-bromotoluene and 4-tolyl-4-methylbenzenesulfonate with vinyl magnesium bromide afforded high yields of 4-methylstyrene (Table 3, entries 25 and 26).

**Table 3:**
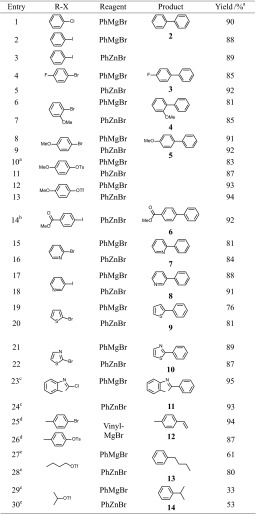
Nickel catalyzed Kumada–Corriu and Negishi cross-coupling of aryl, heteroaryl, and alkyl halides and pseudohalides with organomagnesium and organozinc reagents

Kumada–Corriu coupling: substrate (1 mmol), catalyst (2 mol%, 2 µmol), 3 cm^3^ THF, and organomagnesium reagent (1.3 mmol) stirred for 6 h at r.t.; Negishi coupling: substrate (1 mmol), catalyst (2 mol%, 2 µmol), LiCl (1.3 mmol), 3 cm^3^ THF, and organozinc reagent (1.3 mmol) stirred for 6 h at 60 °C

^a^Yield of pure isolated product after column chromatography

^b^Reaction time 16 h

^c^Reaction time 4 h

^d^Vinyl magnesium bromide (1 M in THF)

^e^Catalyst (5 mol%, 5 µmol), addition at −10 °C and stirring for 2 h before heating to r.t. and 60 °C, respectively

## Conclusion

In conclusion, we have developed a convenient and practical protocol for the Kumada–Corriu and Negishi cross-coupling reaction of challenging electrophiles such as aryl, heteroaryl, and alkyl halides and pseudohalides catalyzed by an air-stable Ni(II) PNP pincer complex based on a triazine scaffold.

## Experimental

All manipulations were performed under an inert atmosphere of argon using Schlenk techniques or in a MBraun inert-gas glovebox. The solvents were purified according to standard procedures [34].The deuterated solvents were purchased from Aldrich and dried over 4 Å molecular sieves. The complexes were prepared according to the literature [27, 35]. All organic substrates, organomagnesium, and organozinc reagents are known compounds and were used as obtained from commercial sources. Room temperature ^1^H and ^13^C{^1^H} NMR spectra were recorded on Bruker AVANCE-250 and AVANCE-400 spectrometers. ^1^H and ^13^C{^1^H} NMR spectra were referenced internally to residual protio-solvent, and solvent resonances, respectively, and are reported relative to tetramethylsilane (*δ* = 0 ppm). A Biotage Initiator Classic system with auto sampler was used for the microwave reaction. As reaction vessel screw cap vials were used. Column chromatography was performed on silica gel 60 from Merck. For thin layer chromatography (TLC) aluminum backed silica gel was used.

### General procedure for the Kumada–Corriu cross-coupling

Substrate (0.1 mmol) and catalyst (2 mol%, 2 µmol) were mixed in 3 cm^3^ THF, organomagnesium reagent (1.3 mmol) was added at room temperature. After 6 h at 60 °C, 1.5 cm^3^ NaCl solution (15%) was added carefully, the organic layer was dried over MgSO_4_, evaporated and purified via silica column chromatography.

### General procedure for the Negishi cross-coupling

Substrate (0.1 mmol) and catalyst (2 mol%, 2 µmol) were mixed in 3 cm^3^ THF, organozinc reagent (1.3 mmol), and LiCl (1.3 mmol) were added at room temperature. After 6 h at 60 °C, the solution was allowed to reach room temperature. NaCl solution (15%, 1 cm^3^) was added carefully, the organic layer was dried over MgSO_4_, evaporated and purified via silica column chromatography.

## Electronic supplementary material

Below is the link to the electronic supplementary material.

Supplementary material 1 (DOCX 45 kb)
