# Supplementary material for: A triazine-based Ni(II) PNP pincer complex as catalyst for Kumada–Corriu and Negishi cross-coupling reactions
Source: Monatsh Chem. 2016 Dec 9;148(1):105–9. doi: 10.1007/s00706-016-1878-4 (PMC5225227; doi:10.1007/s00706-016-1878-4)
Supplement: Supplementary file 1 — Supplementary material 1 (DOCX 45 kb) [file 706_2016_1878_MOESM1_ESM.docx]

**A triazine-based Ni(II) PNP pincer complex as catalyst for Kumada-Corriu and Negishi cross coupling reactions**

**Mathias Mastalir ● Karl Kirchner**

**Supplementary Material**

**Compound charachterization**

**Experimental**

All manipulations were performed under an inert atmosphere of argon by using Schlenk techniques or in a MBraun inert-gas glovebox. The solvents were purified according to standard procedures [34].The deuterated solvents were purchased from Aldrich and dried over 4 Å molecular sieves. The complexes were prepared according to the literature [27]. All organic substrates, organomagnesium and organozinc reagents are known compounds and were used as obtained from commercial sources. Room temperature ^1^H and ^13^C{^1^H} NMR spectra were recorded on Bruker AVANCE-250 and AVANCE-400 spectrometers. ^1^H and ^13^C{^1^H} NMR spectra were referenced internally to residual protio-solvent, and solvent resonances, respectively, and are reported relative to tetramethylsilane (σ = 0 ppm). A Biotage Initiator Classic system with auto sampler was used for the microwave reaction. As reaction vessel screw cap vials were used. Column chromatography was performed on silica gel 60 from Merck. For thin layer chromatography (TLC) aluminum backed silica gel was used.

*4-Methylbiphenyl (****1****)*

Purified by chromatography on SiO_2_ (*n-*hexane); yield as colorless crystals. ^1^H NMR (δ, CD_2_Cl_2_): 7.77 – 7.40 (m, 9H, PhH), 2.35 (s, 3H, CH_3_). ^13^C{^1^H} NMR (δ, CD_2_Cl_2_): 141.2, 138.3, 137.3, 131.0, 129.6, 128.1, 127.1, 127.0, 21.0.

*Biphenyl (****2****)*

Purified by chromatography on SiO_2_ (*n-*hexane); yield as colorless solid. ^1^H NMR (δ, CD_2_Cl_2_): 7.70-7.67 (m, 4H, PhH), 7.60-7.39 (m, 6H, PhH). ^13^C{^1^H} NMR (δ, CD_2_Cl_2_): 140.6, 128.8 (br), 127.4, 127.1.

*4-Fluorobiphenyl (****3****)*

Purified by chromatography on SiO_2_ (*n-*hexane); yield as colorless solid. ^1^H NMR (δ, CD_2_Cl_2_): 7.62-7.56 (m, 4H, PhH), 7.48-7.33 (m, 3H, PhH), 7.19-7.12 (m, 2H, PhH). ^13^C{^1^H} NMR (δ, CD_2_Cl_2_): 164.4, 160.5, 140.1, 137.4, 137.4, 137.3, 134.8, 128.8, 128.8, 128.6, 127.3, 126.9, 115.7, 115.4.

*2-Methoxybiphenyl (****4****)*

Purified by chromatography on SiO_2_ (*n-*hexane); yield as colorless oil. ^1^H NMR (δ, CD_2_Cl_2_): 7.61-7.37 (m, 6H, PhH), 7.13-7.08 (m, 3H, PhH), 3.73 (s, 3H, CH_3_). ^13^C{^1^H} NMR (δ, CD_2_Cl_2_): 156.7, 138.8, 130.8, 130.7, 129.6, 128.7, 128.0, 126.9, 120.8, 111.3, 55.4.

*4-Methoxybiphenyl (****5****)*

Purified by chromatography on SiO_2_ (*n-*hexane); yield as colorless solid. ^1^H NMR (δ, CD_2_Cl_2_): 7.63 (m, 3H, PhH), 7.47 (t, *J* = 7.9 Hz, 2H, PhH), 7.35 (t, *J* = 7.2 Hz, 2H, PhH), 7.03 (d, *J* = 8.3 Hz, 2H, PhH), 3.88 (s, 3H, CH_3_). ^13^C{^1^H} NMR (δ, CD_2_Cl_2_): 159.2, 140.1, 133.5, 128.7, 128.1, 126.7, 126.6, 114.2, 55.3.

*Methyl 4-Biphenylcarboxylate (****6****)*

Purified by chromatography on SiO_2_ (*n-*hexane); yield as colorless crystals. ^1^H NMR (δ, CDCl_3_): 8.14 (dt, J = 1.9 Hz, J = 8.5 Hz, 2H, PhH), 7.71-7.63 (m, 4H, PhH), 7.53-7.39 (m, 3H, PhH), 3.97 (s, 3H, CH_3_). ^13^C{^1^H} NMR (δ, CDCl_3_): 167.0, 145.7, 140.0, 130.1, 128.9, 128.2, 127.3, 127.1, 52.1.

*2-Phenylpyridine (****7****)*

Purified by chromatography on SiO_2_ (*n-*hexane/EtOAc, 8:2); yield as yellow oil. ^1^H NMR (δ, CD_2_Cl_2_): 8.79-8.77 (m, 1H, ArH), 8.17-8.13 (m, 2H, ArH), 7.81-7.80 (m, 2H, ArH), 7.56-7.54 (m, 3H, ArH), 7.30-7.26 (m, 1H, ArH).; ^13^C{^1^H} NMR (δ, CD_2_Cl_2_): 157.1, 149.7, 139.5, 136.7, 129.0, 128.7, 126.9, 122.2, 120.3.

*3-Phenylpyridine (****8****)*

Purified by chromatography on SiO_2_ (*n-*hexane/EtOAc, 8:2); yield as colorless oil. ^1^H NMR (δ, CD_2_Cl_2_): 9.05-9.04 (m, 1H, PyH), 8.79-8.76 (m, 1H, PyH), 8.19-8.16 (m, 1H, PyH), 8.05-8.00 (m, 1H, ArH), 7.88 (d, J = 7.6 Hz, 1H, ArH), 8.60 (d, J = 3.9 Hz, 1H, ArH), 8.86 (s, 1H, ArH). ^13^C{^1^H} NMR (δ, CD_2_Cl_2_): 146.7, 146.5, 137.3, 135.7, 133.6, 129.2, 128.5, 127.2, 124.2.

*2-Phenylthiophene (****9****)*

Purified by chromatography on SiO_2_ (*n-*hexane); yield as colorless oil. ^1^H NMR (δ, CD_2_Cl_2_): 7.75-7.69 (m, 2H, ArH), 7.54-7.37 (m, 5H, ArH), 7.20-7.16 (m, 1H, ThH). ^13^C{^1^H} NMR (δ, CD_2_Cl_2_): 144.4, 134.4, 129.0, 127.6, 127.1, 125.9, 124.9, 123.2.

*1-Phenylthiazole (****10****)*

Purified by chromatography on SiO_2_ (*n-*hexane /Et_2_O 9:1); yield as yellow oil. ^1^H NMR (δ, CD_2_Cl_2_): 8.10-8.06 (m, 2H, PhH), 7.94 (d, J = 3.3 Hz, 1H, ThzH), 7.53-7.50 (m, 3H, PhH), 7.41-7.38 (m, 1H, ThzH). ^13^C{^1^H} NMR (δ, CD_2_Cl_2_): 168.2, 143.7, 133.8, 130.0, 129.0, 126.6, 119.0.

*1-Phenylbenzoxazole (****11****)*

Purified by chromatography on SiO_2_ (*n-*hexane/Et_2_O 9:1); yield as pale yellow solid. ^1^H NMR (δ, CD_2_Cl_2_): 8.34-8.30 (m, 2H, PhH), 7.85-7.81 (m, 1H, PhH), 7.65-7.54 (m, 3H, PhH), 7.45-7.40 (m, 3H, PhH). ^13^C{^1^H} NMR (δ, CD_2_Cl_2_): 163.0, 150.9, 142.3, 131.5, 128.9, 127.6, 127.3, 125.1, 124.5, 120.0, 110.6.

*4-Methylstyrene (****12****)*

Purified by chromatography on SiO_2_ (*n-*hexane); yield as colorless liquid. ^1^H NMR (δ, CD_2_Cl_2_): 7.47 ( d, J = 8.4 Hz, 2H, PhH), 7.30 (d, J = 8.3 Hz, 2H, PhH), 6.86 (dd, J = 17.6 Hz, 10.9 Hz, 1H, CH=CH_2_), 5.88 (d, J = 17.5 Hz, 1H, CH=CH_2_), 5.35 (d, J = 10.8 Hz, 1H, CH=CH_2_), 2.49 (s, 3H, CH_3_); ^13^C NMR (δ, CD_2_Cl_2_): 137.8, 136.9, 135.0, 129.3, 126.2, 112.6, 21.0.

*n-Butylbenzene (****13****)*

Purified by chromatography on SiO_2_ (*n-*hexane); yield as colorless liquid. ^1^H NMR (δ, CD_2_Cl_2_): 7.35-7.23 (m, 5H, PhH), 2.67 (t, J = 7.7 Hz, 2H, CH_2_), 1.72-1.60 (m, 2H, CH_2_), 1.50-1.35 (m, 2H, CH_2_), 1.00 (t, J = 7.1 Hz, 3H, CH_3_). ^13^C{^1^H} NMR (δ, CD_2_Cl_2_): 143.1, 128.4, 128.2, 35.7, 33.8, 22.4, 13.8.

*Cumene (****14****)*

Purified by chromatography on SiO_2_ (*n-*hexane); yield as colorless liquid. ^1^H NMR (δ, CD_2_Cl_2_): 7.39-7.24 (m, PhH), 3.04-2.93 (m, CH), 1.33 (d, J = 7.68 Hz, CH_3_). ^13^C{^1^H} NMR (δ, CD_2_Cl_2_): 149.0, 128.3, 126.4, 125.8, 34.2, 23.9.
